# Age-Related NAFLD: The Use of Probiotics as a Supportive Therapeutic Intervention

**DOI:** 10.3390/cells11182827

**Published:** 2022-09-10

**Authors:** Lucrezia Irene Maria Campagnoli, Nicoletta Marchesi, Mariapia Vairetti, Alessia Pascale, Andrea Ferrigno, Annalisa Barbieri

**Affiliations:** 1 Department of Drug Sciences, Pharmacology Section, University of Pavia, 27100 Pavia, Italy; 2 Unit of Cellular and Molecular Pharmacology and Toxicology, Department of Internal Medicine and Therapeutics, University of Pavia, 27100 Pavia, Italy

**Keywords:** liver, age-related disease, NAFLD, microbiota, therapeutic strategies

## Abstract

Human aging, a natural process characterized by structural and physiological changes, leads to alterations of homeostatic mechanisms, decline of biological functions, and subsequently, the organism becomes vulnerable to external stress or damage. In fact, the elderly population is prone to develop diseases due to deterioration of physiological and biological systems. With aging, the production of reactive oxygen species (ROS) increases, and this causes lipid, protein, and DNA damage, leading to cellular dysfunction and altered cellular processes. Indeed, oxidative stress plays a key role in the pathogenesis of several chronic disorders, including hepatic diseases, such as non-alcoholic fatty liver disease (NAFLD). NAFLD, the most common liver disorder in the Western world, is characterized by intrahepatic lipid accumulation; is highly prevalent in the aging population; and is closely associated with obesity, insulin resistance, hypertension, and dyslipidemia. Among the risk factors involved in the pathogenesis of NAFLD, the dysbiotic gut microbiota plays an essential role, leading to low-grade chronic inflammation, oxidative stress, and production of various toxic metabolites. The intestinal microbiota is a dynamic ecosystem of microbes involved in the maintenance of physiological homeostasis; the alteration of its composition and function, during aging, is implicated in different liver diseases. Therefore, gut microbiota restoration might be a complementary approach for treating NAFLD. The administration of probiotics, which can relieve oxidative stress and elicit several anti-aging properties, could be a strategy to modify the composition and restore a healthy gut microbiota. Indeed, probiotics could represent a valid supplement to prevent and/or help treating some diseases, such as NAFLD, thus improving the already available pharmacological intervention. Moreover, in aging, intervention of prebiotics and fecal microbiota transplantation, as well as probiotics, will provide novel therapeutic approaches. However, the relevant research is limited, and several scientific research works need to be done in the near future to confirm their efficacy.

## 1. Introduction

The so-called gut microbiota (GM) is constituted by numerous different populations of microorganisms (bacteria, archaea, fungi, and viruses) that reside in the gastrointestinal tract of mammals. In recent years, a significant interest in the intestinal microbiota has spread, as it is considered one of the key factors contributing to the maintenance of physiological intestinal homeostasis, the protection against pathogens, and the modulation of the immune system. All these important functions make the GM a fundamental system able to regulate the host’s health [1,2]. Many researches on GM composition, conducted both in animals and humans, have highlighted its involvement in the onset and progression of several disorders, including neurodegenerative; cardiovascular; gastrointestinal; and metabolic diseases, such as obesity, type 2 diabetes, and non-alcoholic fatty liver disease (NAFLD) [3].

The progressive degeneration of the tissues, with consequent alteration of organs’ structure and function, and the loss of homeostasis, make the elderly people more prone to develop diseases [4,5,6]. During aging, it is widely reported that the increased imbalance between reactive oxygen species (ROS) production and antioxidant enzymes expression leads to the onset of oxidative stress (OS), with consequent damage to proteins, DNA, and cellular organelles [7]. Specifically, in the gut, OS, together with a sedentary lifestyle, changes in diet, and administration of drugs, causes GM dysbiosis, which contributes to the increase in intestinal permeability, resulting in the release of bacteria, endotoxins, and pro-oxidants into the systemic circulation. Ultimately, all these factors contribute to the development of hepatic diseases, such as NAFLD [8]. Currently, NAFLD is considered the most common chronic liver disease in the Western world and it is characterized by an excessive intrahepatic fat accumulation, and strongly associated with obesity, hypertension, and insulin resistance [9]. The pathogenesis of NAFLD is not completely understood, but the most accredited hypothesis is the interaction among environmental factors (such as a hypercaloric diet), GM changes, sedentary lifestyle, and genetic predisposition [10]. Over time, NAFLD can become non-alcoholic steatohepatitis (NASH), and eventually progress into fibrosis, cirrhosis, and hepatocellular carcinoma [11]. In order to block the progression of NAFLD, thus improving the elderly’s health, the prevention of the disease is important. The use of probiotics, which are alive microorganisms with numerous health benefits, could be a valid strategy, thanks to their ability to restore the GM and relieve oxidative stress [12].

This review aims to underline the possible factors causing GM dysbiosis and intestinal permeability disruption in elderly people, focusing above all on OS, with particular attention to the association between an altered GM and the development of NAFLD. We also discuss the NAFLD-associated GM signatures and the use of probiotics as a potential therapeutic strategy to restore GM to a healthy condition and counteract NAFLD progression.

## 2. Non-Alcoholic Fatty Liver Disease and Non-Alcoholic Steatohepatitis

Non-alcoholic fatty liver disease is an umbrella term including simple steatosis or non-alcoholic fatty liver (NAFL) and its progression into non-alcoholic steatohepatitis. NAFLD is the hepatic manifestation of the “metabolic syndrome”, which also comprises dyslipidemia, hypertension, insulin resistance, and diabetes [13,14]. Recently, the term NAFDL has been proposed to be replaced by the more generic definition of metabolic-associated fatty liver disease (MAFLD), even though, in general, the traditional nomenclature is still preferred by the majority of experts, mainly because many clinical trials are currently specifically targeting NASH [15]. About a quarter of the world population suffers from NAFLD [16], with rates exceeding 43% in patients with metabolic syndrome [17]. Progression into NASH has been observed in about 10% of patients suffering from NAFLD, and more commonly in patients suffering from diabetes (37.7%), who also present the highest prevalence rate of NAFL (55.5%) [18]. In NASH, the hepatic fat deposition is accompanied by an increased free fatty acid oxidation and mitochondrial dysfunction, leading to a chronic inflammatory state, which in turn can lead to high risk of fibrosis, cirrhosis, and hepatocellular carcinoma development [11,19]. Traditionally, two main “hits” were believed to be involved in NAFLD pathogenesis, being the first intrahepatic fat accumulation triggered by a sedentary lifestyle, bad nutritional habits, and insulin resistance [20], and the second a lipid-induced over-production of ROS [21]. The two-hit hypothesis, by a general consensus, is now considered too simplistic and a “multiple-hit hypothesis” has been proposed instead [11]. The multiple-hit hypothesis has been described as an “integrated response” of the organism to the combination of hypercaloric nutrition and sedentary lifestyle in a genetically predisposed host, leading to metabolic syndrome and obesity [22]. These events are accompanied by insulin resistance in the muscle in response to the increased levels of circulating free fatty acids, leading to an increase in hepatic de novo lipogenesis (DNL) and an imbalance in adipose tissue lipolysis, resulting in higher levels of circulating fatty acids conveyed to the liver [22]. Insulin resistance also contributes to the release of adipokines and inflammatory cytokines from the adipose tissue [23]. Aging affects the process of de novo lipogenesis (DNL) mostly through changes in systemic mediators such as insulin and leptin; in fact, aging is an insulin- and leptin-resistant state [24]. Many factors contribute to insulin resistance in aging, including an increase in body adiposity and visceral fat, increased adipose tissue inflammation, an increase in circulating cytokines, sedentary life style, and changes in growth hormone/insulin-like-growth-factor I (GH-IGF) axis [25]. Insulin resistance has been shown to induce an increase in the percent contribution of DNL to hepatic lipid accumulation [26]. Transcription factors sterol regulatory element-binding protein (SREBP)-1c and carbohydrate-responsive element-binding protein (ChREBP)-1, driven by insulin and glucose respectively, play a crucial role in stimulating DNL in the hepatocytes through an increase in the transcription of rate-limiting DNL enzymes such as fatty acid synthase (FAS), stearoyl-CoA desaturase-1 (SCD1), and acyl coA carboxylase (ACC) [27]. Similarly to insulin resistance, in aging also serum leptin levels are increased, along with a paradoxical lack of effects due to multiple causes, including receptor desensitization, mutations in the genes encoding leptin and its receptors as well as proteins involved in self-regulation of leptin synthesis, and changes in blood–brain barrier permeability [28,29]. Leptin serves as the “satiety signal”, acting primarily at the level of the hypothalamus to decrease appetite; so, reduced leptin levels or leptin resistance result in a higher food intake [30]. In addition to its central role, leptin may have a direct action on DNL; receptors for leptin have also been found in peripheral tissues including liver [31], so changes in leptin signaling may also result in direct DNL enzymes positive modulation [24]. In the liver, excessive fat accumulation leads to lipotoxicity, a condition promoting oxidative stress and affecting mitochondrial and endoplasmic reticulum physiological functions [32]. Altogether, these processes lead to hepatic chronic inflammation accompanied by cell death, hepatic stellate cell (HSC) activation, and fibrosis. However, the original assumption that steatosis always precedes inflammation is not always correct; in fact, NASH can also be the initial hepatic injury: it is the timing and the combination of insults that determine whether steatosis or NASH will occur first [33]. Recently, a deficit of lipophagy has been identified as a further contributor to lipid overaccumulation in NAFLD pathogenesis [34]. Lipophagy is a highly regulated step process that consists of: (1) protein-mediated sequestration of lipid droplets within cytosolic vesicles and formation of a phagosome; (2) transport of a phagosome to a lysosome and formation of the autophagolysosome; and (3) lipid degradation by lysosomal lipases [35]. Many proteins are involved in this process. The cargo adapter p62 is essential as it connects the lipidic cargo with autophagosomes; elevated P62 levels usually are a marker or decreased autophagy. LC3-II, a protein that targets to the elongated autophagosome membrane, is degraded by lysosomal proteases; therefore, the increase in LC3-II indicates its impaired turnover [36]. Both P62 and LC3-II proteins were found to accumulate in high-fat diet-fed C57BL/6J male mice and high-fat/high-glucose cultured Huh7 cells [35]. In addition, in NAFLD patients, lipid droplet-loaded lysosomes and P62/sequestosome (SQSTM)1 clusters were associated with NAFLD activity score (NAS) and fibrosis stage, respectively, as well as expression levels of lysosomal genes and autophagy-related genes, showing that impaired autophagy is associated with features of advanced disease [35].

## 3. Gut Microbiota and Oxidative Stress

The human microbiota consists of a wide range of microorganisms that reside in different parts of the body, including the skin and the gastrointestinal, genitourinary, and respiratory tracts [37]. In addition to these body districts, the urethra and the mammary glands have their own microbes [38]. The GM is a complex and dynamic ecosystem of trillions of commensal microorganisms, including different communities of bacteria and some members of archaea, fungi, and viruses, which live in the gastrointestinal tract and give rise to a mutual relationship with the host [39,40]. The colonization of the gastrointestinal tract by bacteria begins in utero, via the placenta and the mother’s amniotic fluid [41], while after birth, the mode of delivery (natural or cesarean), feeding (breastfeeding or artificial), ingestion of antibiotics or probiotics during the early days of life, genetics, and environmental factors influence the composition of GM [39,42]. The *Bifidobacterium* mainly dominates the microbiota profile of infants, which can continually change during the first 3 years of life [43] according to a variety of factors, such as nutrition, geographic distribution [44], genetic background, and immunological stimuli [45]. After 3 years of age, the GM acquires a more complex adult pattern that is relatively stable throughout adulthood [37]. Namely, the GM of healthy adults is composed of anaerobic bacteria, most of which (more than 90%) belong to the phyla of *Bacteroidetes* (*Bacteroides*, *Prevotella*, and *Porphyromonas*) and *Firmicutes* (*Clostridia*), followed by a small percentage (1–8%) of *Actinobacteria* (*Bifidobacterium*), *Proteobacteria*, and *Verrucomicrobia* [46,47]. The GM is necessary for the human’s health; indeed, it can modulate innate and adaptive immune responses, regulate cellular growth, and preserve epithelial barrier function [48]. Furthermore, the GM is also involved in glucose and lipid metabolism, energy balance, detoxification, vitamin K synthesis, and production of short-chain fatty acids (SCFAs; acetate, propionate, and butyrate) [45,47].

**Figure 1:**
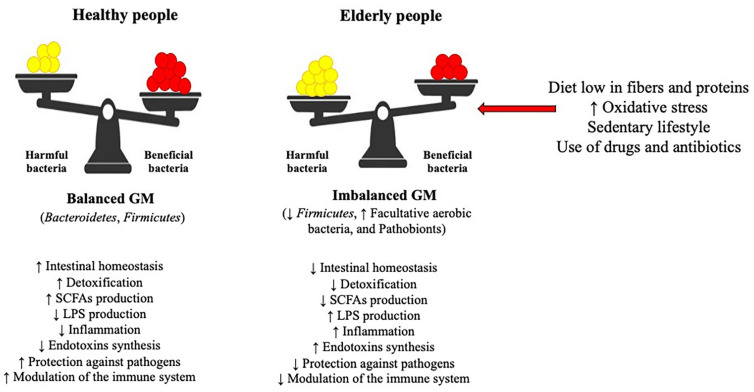
Effects of an altered gut microbiota (GM) in elderly people. A diet low in fibers and proteins, an increase in oxidative stress, a sedentary lifestyle, and an intake of drugs and antibiotics lead to an alteration of the intestinal microbiota, with a reduced production of SCFAs and an increase in LPS, resulting in gut inflammation and development of metabolic diseases. Abbreviations: SCFAs: shot-chain fatty acids; LPS: lipopolysaccharides.

On the whole, based on its composition, the GM is described as an organ with a high degree of variability and heterogeneity, able to change and adapt to the needs of the host’s human body [37]. As a result, each person’s GM has a unique composition that differs from others [49]. Nevertheless, the GM composition changes drastically as people age due to various factors, including lifestyle, dietary habits, stress, use of antibiotics and drugs, and environmental stimuli [39,50] (Figure 1). This event results in damage and loss of the intestinal homeostasis [51], and thus, in the elderly, the GM acquires specific features such as fewer beneficial bacteria, changes in dominant gut species (reduction of *Firmicutes* and increase in facultative aerobic bacteria), and proliferation of pathobionts proliferation (streptococci, staphylococci, enterobacteria, and enterococci), which are responsible for the onset in the gut of an inflammatory state in the gut [37,52,53]. Further, the dysbiotic microbiota is no longer able to perform its primary beneficial functions, thus leading to the production of toxic metabolites and to inflammation, which in turn cause the development of a variety of metabolic diseases, such as hypercholesterolemia, diabetes, obesity, NAFLD, and its progression into NASH [54,55].

Since elderly people have difficulties in swelling and chewing, in association with a decreased digestive motility [56], nutrition plays a key role in changing the GM profile [57]. A diet lacking in fibers and proteins, for example, as well as vitamin D and calcium deficiency, can alter the composition of GM, [58,59]. Moreover, the consumption of plant-based proteins, animal-based proteins, inulin, olive oil, and omega-3 polyunsaturated fatty acids (PUFA) can also modulate GM [60]. Besides diet, GM dysbiosis can as well be caused by oxidative stress and treatment with drugs aimed at targeting human cells rather than microorganisms, such as antidiabetics (metformin), proton pump inhibitors (PPIs), nonsteroidal anti-inflammatory drugs (NSAIDs), and atypical antipsychotics (AAPs) [61,62].

The accumulation of ROS produced by cellular metabolic and respiratory processes is recognized as one of the causes that promotes aging [63]. Namely, ROS and reactive nitrogen species (RNS) are essential for cellular proliferation and differentiation, cytokines release, metabolism, and immune response. They are naturally produced by the organism’s cells at low levels [7]. Under physiological conditions, the organism has several antioxidative defense mechanisms, including enzymes (catalase, glutathione peroxidase, and superoxide dismutase) and antioxidants (such as vitamin C, vitamin E, uric acid, carotenoids, and flavonoids), which can protect it against oxidant species; instead, as people get older, there is a cellular imbalance between these defenses and ROS generation, in favor of oxidants, resulting in OS [7,64]. OS causes molecular and cellular damage, particularly to proteins, lipids, DNA, and organelles [7,65], thus contributing to uncontrolled proliferation, inflammation, and cell death through apoptosis [66].

During aging, the alteration of cellular macromolecules, as well as the dysfunction at the level of mitochondria, which represents the primary source of energy, and the subsequent further production of ROS, eventually lead to the onset of age-related disorders [64].

Specifically, at the intestinal level, the continuous exposure of the mucosa to oxidants derived from diet and bacteria results in excessive production of ROS and the consequent onset of oxidative stress [66]. OS may disrupt colonic epithelial tight junctions, subsequently increasing the intestinal mucosa permeability, thus leading to a phenomenon known as “leaky gut” syndrome. This condition is characterized by the translocation of pro-oxidants and antigens, such as lipopolysaccharides (LPS), bacteria, and their endotoxins into the systemic circulation, where they reach various target organs [40,46], resulting in several pathological conditions, including metabolic disorders and infectious and systemic diseases (such as cardiac, neurodegenerative, and neoplastic) [40,67].

As mentioned before, whereas the composition of GM seems to be directly associated with ROS production in the intestine [68], other authors [60], conversely revealed that the abundance and composition of the GM may influence the intestinal production of ROS. In fact, the consumption of probiotic bacteria and antioxidant nutrients able to change GM may lower ROS production by inhibiting pro-oxidant enzymes and stimulating antioxidant enzymes and related pathways [60]. Furthermore, the GM itself can produce antioxidant molecules (glutathione, butyrate, and folate) able to protect the gut from toxins and ROS [69].

**Figure 2:**
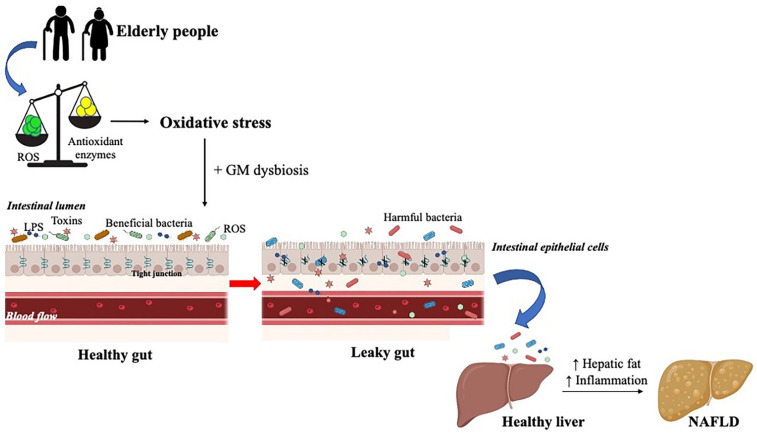
”Leaky gut” syndrome contributes to the onset of NAFLD. In the elderly, the imbalance between the formation of ROS and the presence of antioxidant enzymes leads to oxidative stress, which, at the intestinal level contributes to gut microbiota (GM) dysbiosis. These two conditions disrupt the tight junctions of the epithelial cells, resulting in the translocation of endotoxins, lipopolysaccharides (LPS), harmful bacteria, and ROS from the intestinal lumen into the blood circulation where they are taken up by the liver and cause an increase in lipogenesis and inflammation, finally resulting in the development of this hepatic disease.

Finally, given that an imbalance between oxygen species generation and antioxidant defenses causes intestinal damage, an excessive amount of ROS and RNS leads to an elevated cellular oxidative stress, contributing to the GM dysbiosis, which favors several gastrointestinal conditions, such as inflammation and metabolic disorders, like NAFLD [60]. In fact, the alteration of the intestinal microbiota is an important factor that contributes to the pathogenesis of NAFLD and its progression into NASH [54]. In particular, dysbiosis and oxidative stress lead to a dysregulation of intestinal permeability, resulting in the release of endotoxins, and microbiota metabolites, derived from saccharolytic and proteolytic fermentation, at the level of the liver, with a consequent increase in the accumulation of hepatic fat and inflammation [8], typical signs of this disease. Over time, if these conditions persist, NAFLD progresses into NASH, the more severe form, characterized by hepatocellular injury, chronic inflammation, and fibrosis [70] (Figure 2).

## 4. Gut Microbiota and NAFLD Development in Animal Models

In the last decades, fecal transplantation experiments in mice have provided a growing body of evidence about a causal role between GM alterations and NAFLD/NASH development [71,72]. GM was studied in various animal models of NAFLD, and its alteration was found to be associated with NAFLD genesis and progression. Adult germ-free mice fed with a regular diet, when exposed to a microbiota harvested from conventionally raised animals, showed a 60% increase in body fat content and the insurgence of insulin resistance, possibly due to the increase in the absorption of monosaccharides from the gut lumen, resulting in the induction of hepatic de novo lipogenesis [73]. Similarly, wild-type germ-free mice fed with a Western-style, high-fat, sugar-rich diet, are less prone to develop steatosis when compared to animals raised in conventional conditions [74,75]. On the contrary, steatosis develops regularly in germ-free knockout (KO) mice lacking fasting-induced adipose factor (FIAF), a circulating lipoprotein lipase inhibitor normally suppressed by GM, suggesting that FIAF is a mediator of microbial-regulated energy storage [74]. Recognizing the role of GM in the development of NAFLD also implies the concept that NAFLD is potentially a transmissible process [76]. In fact, germ-free mice colonized with the cecal content collected from donors either responders or non-responders to a high-fat diet developed symptoms comparable to the respective donor when fed with the same diet. In other words, germ-free mice receiving intestinal microbiota from responder mice developed macrosteatosis and hyperglycemia; differently, mice receiving intestinal microbiota from non-responder mice do not develop NAFLD when fed with a high-fat diet [77]. A similar effect was seen in mice colonized with human GM from healthy individuals or NAFLD patients: mice fed with a high-fat diet developed more severe NAFLD symptoms when receiving the microbiota from NAFLD patients and vice-versa [78]. In a more recent work, quercetin was administered to donor mice fed with a high-fat diet to modulate the microbiota composition; the transplantation of microbiota from quercetin-treated donors in germ-free mice resulted in a protective phenotype against diet-induced NAFLD [79]. Further, in mice fed with a Western diet, a worsening of NASH symptoms was associated with the depletion of G protein-coupled chemokine receptor CX3CR1; the depletion of GM using broad-spectrum antibiotics was also found to protect mice from diet-induced NASH, similarly to what was demonstrated in germ-free mice [80].

The methionine-choline deficient (MCD) diet is another well-established animal model of NAFLD. In this model, choline deficiency affects triglyceride export via very low-density lipoproteins (VLDLs), resulting in hepatic steatosis; in addition, the lack of methionine impairs glutathione synthesis, causing a significant increase in oxidative injury [14]. The MCD diet-induced NAFLD is characterized by hepatic ballooning, marked oxidative stress, chronic inflammation, and fibrosis, without development of hyperglycemia, dyslipidemia, and insulin resistance; therefore, it is more suitable for the study of inflammation and fibrosis [81]. In mice, the administration of MCD diet induces persistent alterations in the GM and impairment of the intestinal barrier [82]. However, unexpectedly, in MCD mice the treatment with broad-spectrum antibiotics, aimed to deplete the microbiota, does not produce the same effect seen in germ-free or antibiotic-treated mice fed with a Western diet, resulting in the aggravation of steatosis, inflammation, a higher histopathological NAFLD activity score (NAS), and a significantly higher liver-to-body weight ratio [83]. In contrast, the microbiota modulation via probiotics has shown beneficial effects both in the high-fat NAFLD model and in the MCD-induced NASH [84], suggesting that in the MCD model, the microbiota preserves its protective activity, which is lost in high-fat and Western diet models of NAFLD. These last works suggest that the microbiota should be seen as both a potential therapeutic agent and a drug target for the treatment of NAFLD.

An alternative NAFLD model consists of the supplementation of high doses of fructose in a regular diet [14]. Interestingly, high fructose supplementation does not necessarily result in body weight gain, but indeed in the increase in liver weight/body weight ratio [75], supporting the newly acquired notion that diet-induced liver steatosis does not necessarily precede body weight gain [85]. Fructose at high doses has been found to be associated with microbiota overgrowth and increased intestinal permeability, leading to an endotoxin-dependent activation of hepatic Kupffer cells. In fact, the suppression of endotoxin-mediated activation of Kupffer cells in toll-like receptor (TLR)-4 mutant mice resulted in the reduction of hepatic triglyceride accumulation by approximately 40% in comparison with fructose-fed wild-type mice [86]. Fructose-induced steatosis is also absent in germ-free mice, confirming that bacterial products such as LPS are required to induce liver steatosis and clearly indicating that the gut microbiota is involved in the pathogenesis of experimental fatty liver disease [75].

Several hypotheses have been formulated as to how the GM may contribute to NAFLD development and progression into NASH. As previously mentioned, they include increased intestinal permeability, leading to an increased absorption by the host of microbially produced toxins and metabolites, such as LPS, trimethylamine N-oxide (TMAO), choline, and ethanol, which trigger inflammation and affect immunity [71]. Infiltrating immune cells such as monocyte-derived macrophages and neutrophil granulocytes, two mediators of the hepatic inflammation during NASH, seem to have a relevant role in the microbiota-mediated worsening of NAFLD; in fact, chemokine receptor antagonists, by inhibiting monocyte recruitment, reduce hepatocyte ballooning, fibrosis, and inflammation in both the Western diet and the MCD diet models [87]. Infiltrating immune cells express high levels of pathogen recognition receptors (PRRs), including the NLR inflammasome family members, designated to recognize toxins released by the microbiota that reach the liver via the portal circulation [88]. Interestingly, in NLRP3 and NLRP6 inflammasome-deficient mice, an unfavorable intestinal microbiota has been linked to a loss of intestinal barrier integrity and increased translocation of toxins of microbial origin into the liver, where they activate hepatic inflammation [89]. These data indicate that translocation of bacterial products from the gut into the liver is part of a highly regulated series of complex interactions among the gut, its microbiota, and the liver, often referred to as the gut–liver axis, and contributing to liver fat accumulation and inflammation in NASH [90].

## 5. Changes in Gut Microbiota in Animal Models of NAFLD

Many preclinical studies have tried to associate specific alterations in GM composition, often referred to as a microbial signature, with NAFLD and NASH development. Prolonged (80 weeks) high-fat diet feeding in mice was associated with an increase in the relative abundance of the *Firmicutes* phylum with respect to the *Bacterioidetes*; at the genus level, an increase in the abundance of *Adercreutzia* (*Actinobacteria*), *Coprococcus* (*Firmicutes*), *Dorea* (*Firmicutes*), and *Ruminococcus* (*Firmicutes*) was observed in mice fed with a high-fat diet in comparison with the low-fat diet group [91]. In germ-free mice colonized with the microbiota from responder and non-responder mice to high-fat diet, NAFLD was positively associated with *Barnesiella* and *Roseburia* (from the *Bacteroidetes* and *Firmicutes* genera, respectively); after 16 weeks of high-fat diet administration, an increase in *Barnesiella* and *Allobaculum* and a decrease in *Lactobacilli* were observed. In general, the *Firmicutes* phylum was more represented in mice developing NAFLD [77]. Overall, the increase in *Firmicutes*/*Bacteroidetes* has been associated with NAFLD progression, even though there is not a complete consensus. In this last regard, in another work, *Firmicutes* and *Verrucomicrobiota* phyla were instead found to be more represented in mice not developing NAFLD and, at the genus level, *Bacteroidia* and *Flavobacteriia* were increased in mice developing NAFLD [79]. The administration of VSL#3, a high-concentration mixture of *Bifidobacteria*, *Lactobacilli*, and *Streptococcus thermophilus* improved liver histology, reduced hepatic total fatty acid content, and decreased serum alanine aminotransferase levels in mice fed with high-fat diet. The histological and biochemical improvement were associated with lower levels of two nuclear factors regulated by tumor necrosis factor (TNF): Jun N-terminal kinase (JNK) and nuclear factor B (NF-B), both involved in the development of insulin resistance [84].

In mice fed with the MCD diet for 2 and 4 weeks, the phylum of *Tenericutes* was more abundant compared with that of the respective control groups, while *Verrucomicrobia* were consistently less abundant. After 2 weeks of MCD diet, a significantly higher abundance of *Firmicutes* and a significantly reduced content of *Proteobacteria* were seen; at 4 weeks, a decrease in *Actinobacteria* was observed. At the family level, *Rikenellaceae*, *Desulfovibrionaceae*, and *Verrucomicrobiaceae* were persistently reduced in the MCD group when compared with the 4-week control group [82]. After 8 weeks, MCD feeding resulted in a strong overall decrease of the microbiota diversity and in a reduction in the potentially probiotic *Lactobacillus*, as well as *Akkermansia*, and an increase in the *Ruminococus*, which has been linked to liver fibrosis [83].

## 6. Association between Gut Microbiota and NAFLD Development in Humans

Several large human studies have investigated a microbial signature possibly predicting the risk of progression from simple steatosis toward more advanced disease stages [76]; however, a certain level of discrepancy was found among studies, with divergent results concerning phylum, family, genus, and species. The phyla of *Firmicutes* and *Bacteroidetes* are the most represented in the gut microbiome; consequently, many animal and human studies focused on the relative abundance of these two groups. Similarly to what had been found in animal studies [77,91,92], it was originally proposed that an increase in the *Firmicutes*-to-*Bacteroidetes* ratio was associated with a higher energy harvest and more severe NAFLD manifestations in obese individuals [93]; however, this notion was challenged by more recent findings [94,95,96]. Specifically, in NAFLD patients, *Firmicutes* were found to be increased in studies by Del Chierico (2017) and Loomba (2017) [97,98], decreased in studies by Wang [99] and Zhu [94], and unaltered in those by Raman (2013) and Alferink [96,100]. *Bacteroidetes* were more represented in NAFLD patients in the studies by Wang [99] and Zhu [94], decreased in the studies by Del Chierico [97] and Shen [95], and unaltered in the studies from Alferink [96]. It has been proposed that using higher phylogenetic levels (i.e., phylum) to distinguish disease states naturally leads to discrepancies; therefore, the studies should focus on lower levels, such as the genus [100]; however, discrepancies have also been found when considering the genus level, with regard to *Prevotella*, *Oscillibacter*, *Bifidobacterium*, *Blautia*, *Lactobacillus*, and *Roseburia* [72]. These discrepancies may originate from the fact that NAFLD is heterogeneous by nature, and the studies often include patients at different stages of disease severity, with compensated or decompensated cirrhosis [72].

Nonetheless, concordant changes were found in patients with NAFLD and NASH, in comparison with healthy individuals. Indeed, the phylum of *Proteobacteria* was increased [95,98,100]; at the family level, *Enterobacteriaceae* were increased [94,95], while *Rikenellaceae* [94,97] and *Ruminococcaceae* [95,100] were decreased; the genera *Faecalibacterium* [94], *Coprococcus* [94,99], and *Anaerosporobacter* [99] were also decreased, while *Dorea* was increased [97,100]. An increase in the genera *Escherichia* and *Peptoniphilus* was specific to NAFLD patients without NASH [94,97], as well as a decrease in *Prevotella* [101].

## 7. Probiotics

According to the Food and Agriculture Organization of the United Nations (FAO)/World Health Organization (WHO), probiotics are defined as “live microorganisms which, when administered in adequate amounts, confer a health benefit on the host” [102]. To be used as probiotics, microorganisms must have certain characteristics. They must be alive, safe, non-pathogenic bacteria, able to cross the gut intestinal tract and survive both in acidic (stomach) and basic (duodenum) pH, as well as be resistant to bile, hydrochloric acid, and pancreatic juice [103]. Furthermore, they should be of human origin, isolated from the mouth, gastrointestinal tract, or feces, and belong to a healthy GM. However, probiotic bacteria from the *Lactobacillus* and *Bifidobacterium* genera, as well as other microorganisms, can be also isolated from fermented milk and related products, such as cheese, and yogurt, as well as from traditional drinks (Yosa, Bosa, Pozol, and Togwa) [104]. In addition to these important features, it is essential that probiotics are endowed with an antimicrobial activity against pathogenic bacteria and a reduced intestinal permeability, which allows them to colonize the gut, as well as the ability to stimulate the immune system, by sending signals to gut immune cells, produce lactic acid, and influence intestinal metabolism [104].

It is known that probiotics have beneficial effects for both humans and animals, as they can promote gastrointestinal tract motility and control the intestinal microbiota, improve lactose tolerance, and lower cholesterol levels [105]. They can also favor the proliferation and differentiation of epithelial cells, and reinforce the intestinal barrier [106]. Furthermore, having a therapeutic role, they can confer benefits to the immune, nervous, and gastrointestinal systems, and prevent some diseases, including obesity, diabetes, cardiovascular, liver, and metabolic disorders, as well as cancer and allergies [107,108]. Additionally, they are a helpful solution to counteract other clinical conditions, such as diarrhea, gastroenteritis, Crohn’s disease, female urogenital infections, and alleviate symptoms due to lactose intolerance [104,109] (Table 1).

Interestingly, probiotics, by possessing anti-aging properties, could also favor longevity. In fact, they can be used to activate antioxidant and immunomodulatory pathways, as well as to prevent some typical signs of aging, such as hair loss and skin wrinkles, and to improve skin elasticity [134,135]. Wen-Yang Lin (2022) [12], by administering a mixture of probiotics (*Bifidobacterium animalis* subsp. *infantis BLI-02*, *Bifidobacterium breve Bv889*, *Bifidobacterium bifidum VDD088*, *Bifidobacterium animalis* subsp. *lactis CP-9*, and *Lactobacillus plantarum PL-02*) to aged mice, demonstrated their antioxidant property, resulting in positive modulation of GM and SCFAs synthesis [12]. Moreover, several clinical trials reported the effect of probiotics supplementation on the GM in elderly people [136,137,138,139]. For instance, two randomized, double-blind, placebo-controlled studies showed that *Bifidobacterium longum Bar33* and *Lactobacillus helveticus Bar13* reduce opportunistic pathogens (*Clostridium cluster XI*, *Clostridium difficile* and *Clostridium perfringens*, *Enterococcus faecium* and *Campylobacter*), while supplementation with *Bifidobacterium longum 46* and *B. longum 2C* increases the number of *Bifidobacterium catenulatum*, *Bifidobacterium bifidum*, and *Bifidobacterium breve* [136,137].

Probiotics exert their health benefits through different mechanisms of action: They can compete with pathogenic bacteria for nutrients and adhesion sites on the intestinal mucosa, inhibit the production of bacterial toxins, fortify the epithelial barrier, and possess antimicrobial properties, such as the ability to produce antimicrobial substances (like bacteriocins and SCFAs), through which they can inhibit the growth of pathogens and restrict their adhesion and access across the barrier. Finally, probiotics can also act as immunomodulators, by reducing proinflammatory cytokines secretion [such as interferon gamma (IFNγ), TNFα, and interleukin 12 (IL-12)] and promoting the expression of anti-inflammatory cytokines (such as IL-10), as well as epithelial cells and T lymphocytes proliferation and differentiation [140,141,142].

In addition to the positive effects mentioned above, supplementation with probiotics can also modulate the GM in humans and animals [107]. In the presence of dysbiosis, probiotics species can restore the correct balance of the gut microbial composition and repress pathogens, by eliciting the production of β-defensin and IgA; moreover, they can favor the expression of anti-inflammatory molecules and improve the integrity of the intestinal barrier, by promoting the production of mucin and preventing the disruption of tight junctions [40,143].

The genera *Lactobacillus* and *Bifidobacterium* are the most commonly used probiotic bacteria, followed by *Streptococcus*, *Escherichia*, *Enterococcus*, and *Bacillus*. Some *Saccharomyces* fungal strains can also be used as probiotics [103] (Table 2).

For instance, treatment with *Lactobacillus rhamnosus GG*, a lactic acid bacterium, has been shown to reduce oxidative stress and inflammation in the intestine, modulate the altered microbiota, as well as restore the gut barrier function [144,145,146]; further, it has been reported that the *Lactobacillus acidophilus* can prevent intestinal inflammation, by reducing the expression of proinflammatory cytokines (IL-6, TNFα, IL-1b, and IL-17) and promoting the production of IL-10, as well as by modulating the GM, favoring the increase of beneficial bacteria [147,148]. Like other *Lactobacilli*, also the treatment with *L. Plantarum* has the potential to change the composition of GM, increase SCFAs levels, and decrease the expression of some inflammatory cytokines (such as TNFα, IL1-β, and IL-6), thus preventing metabolic disorders and gut inflammation [149]. In addition to *Lactobacilli*, bacteria of the genus *Bifidobacterium* (such as *B. bifidum*, *B. breve*, and *B. longum*) can also be used as probiotics to modify and stabilize the composition of GM, to inhibit the growth of pathogenic bacteria and the production of proinflammatory cytokines, and to strengthen the gastrointestinal barrier [107]. Additionally, it has been reported that the probiotic bacterium *Escherichia coli Nissle* can modulate the bacterial population of GM and restore the intestinal homeostasis, by producing human β-defensin 2, which is useful as a barrier against the invasion of pathogens (such as *Salmonella*, *Shigella*, and *Candida*) across the intestinal barrier [150,151]. Further, some yeasts, including *Saccharomyces cerevisiae* and *Saccharomyces boulardii*, are also employed as probiotics. They have the ability to modify the GM microorganisms and reduce inflammation [107,152]. Interestingly, in addition to these classic probiotics, several studies have shown the beneficial role of other bacteria, known as “next-generation probiotics” (NGP), such as *Faecalibacterium prausnitzii* and *A. muciniphila*. For instance, *A. muciniphila*, a Gram-negative anaerobic bacterium, has been demonstrated to be able to reduce gut inflammation and strengthen the intestinal barrier, favoring the synthesis of antimicrobial substances, the thickening of mucus, and the restoration of tight junctions proteins expression [153,154].

As previously mentioned, the GM plays a dominant role in the pathogenesis of NAFLD [155]. Changes in its composition (for instance, an increase in Gram-negative bacteria belonging to *Proteobacteria*, *Escherichia*, and *Enterobacteria* species) increase intestinal permeability, resulting in the translocation of endotoxins and toxic metabolites into the liver, and leading to the production of inflammatory cytokines by Kupffer cells [156,157,158]. Furthermore, dysbiosis can also alter the metabolism of bile acids and choline, and increase the production of endogenous ethanol in the intestine. All these events cause inflammation and OS, which in turn trigger the onset of the disease and eventually its progression into cirrhosis [54].

To date, no specific drugs have yet been approved to treat NAFLD. The current strategies employed to control the disease and its progression include lifestyle changes (diet modifications, exercise, and gradual weight loss), the use of hypoglycemic and antioxidant agents, as well as drugs commonly used to treat diabetes mellitus (such as metformin and thiazolidinediones) [159]. The employment of probiotics may provide a new therapeutic approach for managing and treating liver diseases, like NAFLD. In fact, as previously discussed, it is well known that they are able to restore the GM to a healthy state, by improving the expression of occludins and blocking the invasion of pathogenic bacteria and endotoxins into the intestine, and to reduce hepatic inflammation, by balancing the expression of pro-and anti-inflammatory cytokines [54,160,161]. Further, they can also favor the production of SCFAs, decrease the amount of hepatic triglycerides, and relieve the intestinal OS, by increasing the levels of the enzymes superoxide dismutase (SOD) and plasma glutathione peroxidase (GSH-PH) and reducing the content of malondialdehyde (MDA) [155,162,163].

To conclude, it is widely reported that the GM is altered in elderly people, and dysbiosis is linked to the onset of NAFLD [136,155]. Thus, since changes in GM can contribute to the irregular synthesis of bile acids, resulting in the excessive accumulation of fats in the liver and development of the disease, the use of probiotics to restore GM composition could be effective to modulate bile acids production and manage NAFLD [55].

### 7.1. Preclinical Studies of Probiotic Supplementation in NAFLD

Several animal studies have been conducted to evaluate the possible effects of probiotics on NAFLD development and progression (Table 3). It has been reported that *Lactobacillus plantarum NCU116* and *Lactobacillus plantarum NA136* could be safe probiotics for NAFLD. Notably, *L. plantarum NCU116* had beneficial effects in NAFLD model rats, by inhibiting inflammation (decrease TNFα and IL-6 expression) and hepatic oxidative stress (increase SOD, GSH-Px, and catalase activities), and by restoring bacteria flora [164], while *Lactobacillus plantarum NA136* could alleviate NAFLD in mice, by increasing nuclear factor erythroid 2-related factor 2 (Nrf2) and AMP-activated protein kinase (AMPK) cascades, resulting in the activation of different antioxidant pathways and regulation of the fatty acid metabolism [165]. Further, it has been shown that *Lactobacillus johnsonii BS15* may prevent NAFLD in obese mice, by improving mitochondrial dysfunction and reducing inflammation and gut permeability [166], while treatment with *Lactobacillus rhamnosus GG* could protect mice and rats from NAFLD, by reducing liver fat accumulation and inflammation (decrease TNFα, IL-1β, and IL-8R mRNA expression) [167], and stimulating sirtuins type 1 (SIRT1)-mediated signaling pathway [168], respectively. In other studies, supplementation with *Bifidobacterium longum* attenuated liver fat accumulation in NAFLD model rats [169]; treatment with a mixture of probiotics (*Bacillus animalis VKB*, *Bacullus animalis VKL*, *Lactobacillus casei IMV B-7280*) modulated the GM composition and reduced cholesterol level, oxidative stress, and weight in obese mice [170]; and administration of *Bifidobacterium infantis, Lactobacillus acidopilus*, and *Bacillus cereus* in rats restored the GM structure, and decreased serum levels of gut-derived bacterial lipopolysaccharide (LPS) and inflammatory cytokines (TNFα and IL-18), and liver toll-like receptor 4 (TLR4)-mRNA [171]. Another study in rats revealed that supplementation with *Clostridium butyricum MIYAIRI 588* could improve NAFLD, by decreasing accumulation of lipids droplets [172]. Finally, treatment with *VSL#3* probiotics alleviated obesity, hepatic steatosis, and insulin resistance, as well as reduced inflammation, downregulating the activation of TNFα/inhibitor of nuclear factor kappa-B kinase subunit beta (IKK-β) signaling pathway in high-fat diet-fed mice [173]. Moreover, *VSL#3* probiotics may reduce alanine aminotransferase (ALT) levels and hepatic total fatty acid in high-fat diet model mice [174].

In addition to traditional probiotics, the emerging NGP, including, *A. muciniphila*, *F. prausnitzii*, *Bacteroides* spp., and the *Roseburia*, could represent a potential therapeutic strategy for the treatment of NAFLD [155]. For instance, Munukka (2017). reported the ability of *F. prausnitzii* probiotic to improve hepatic health, by decreasing fibrosis, aspartate aminotransferase (AST) and ALT levels, and fat content in liver of high-fat fed mice [175].

### 7.2. Clinical Trials of Probiotic Supplementation in NAFLD

Some human studies have demonstrated the benefits of probiotic supplementation in patients with NAFLD (Table 3). It has been demonstrated that administration of conventional yogurt, fermented by *Lactobacillus delbrueckii* ssp. *bulgaricus* and *Streptococcus thermophiles*, as well as the supplementation of a mixture of six probiotics (*L. acidophilus*, *L. rhamnosus*, *L. paracasei*, *Pediococcus pentosaceus*, *B. lactis*, and *B. breve*) can have beneficial effects on patients with NAFLD, by modifying the GM composition, and reducing inflammation (decrease TNFα expression) and lipid metabolism (decrease total cholesterol and triglycerides) [176,177]. A randomized, double-blind, placebo-controlled clinical trial showed that multistrain probiotic supplementation can decrease insulin, insulin resistance, TNFα, and IL-6 in patients with NAFLD [178]; further, in the same line, treatment with *Lactobacillus bulgaricus* and *Streptococcus thermophilus* can decrease ALT and AST activity, and gamma glutamyl transferase (GGT) levels in NAFLD patients [179]. Another randomized, double-blind, placebo-controlled clinical trial reports that administration of *VSL#3* decreased triglycerides and high-sensitivity C-reactive protein levels, as well as transaminases and GGT activity [180]. Interestingly, Shavakhi et al. demonstrated that treatment with Metformin plus Protexin (*L. acidophilus*, *L. casei*, *L. rhamnosus*, *L. bulgaricus*, *B. breve*, *B. longum*, *Streptococcus thermophilus*) decreases ALT and AST activity, better than Metformin alone in patients with NASH [181]. Finally, the use of a cocktail of 14 probiotic strains, belonging to *Lactobacillus* + *Lactococcus*, *Bifidobacterium*, *Propionibacterium*, and *Acetobacter* genera, could improve hepatic steatosis, by reducing AST and GGT activity, as well as TNFα and IL-6 levels, in NAFLD patients [182].

## 8. Other Therapeutic Options

As widely reported, GM modulation represents a valid approach to manage many diseases, including NAFLD. In addition to probiotics, prebiotics, symbiotics, and the so-called fecal microbiota transplant (FMT) represent other methods used to restore dysbiosis [188,189].

Prebiotics are “non-digestible food ingredients that beneficially affect the host’s health, by selectively stimulating the growth and/or activity of beneficial bacteria in the gastrointestinal tract” [190]. Most of them are non-digestible fibers, such as fructo-oligosaccharides (FOS), galacto-oligosaccharides (GOS), lactulose, inulin, and pectin [191]. They can prevent diarrhea, as well as cancer, modulate the metabolism of the intestinal flora, stimulate mineral adsorption, and have positive effects on lipid metabolism and immunomodulatory properties [192]. In addition, prebiotics can modulate the composition of GM, by promoting the growth of beneficial microorganisms and reducing the number of Gram-negative bacteria [193,194,195]. Some evidence showed that prebiotic supplementation can prevent NAFLD development and progression [196,197]. Studies report that prebiotic fructo-oligosaccharides restored normal gastrointestinal microflora and intestinal epithelial barrier function, and decreased steatohepatitis in NASH model mice, while lactulose improved hepatic inflammation and decreased ALT and AST serum level in NASH model rats [198,199]. Moreover, a randomized, double-blind, placebo-controlled clinical trial reported that *Chlorella vulgaris* can decrease serum glucose level and improve liver function and lipid profile in NAFLD patients [200]; further, in the same line, Javadi (2017) showed that prebiotic inulin reduces AST and ALT levels, compared to placebo. However, they found no significant changes in the grade of fatty liver [201]. Finally, administration of oligofructose decreased ALT, AST, and insulin serum level in patients with NASH [197]. Interestingly, some studies report the effects of prebiotics on the GM in elderly people [202,203,204]. Two randomized, double-blind, placebo-controlled clinical trials show that galacto-oligosaccharides mixture (B-GOS) increased the number of beneficial bacteria, especially *Bifidobacteria* [202,203], as well as GOS supplementation [204].

*Symbiotics* are the combination of probiotics and prebiotics, where prebiotics favor the proliferation of healthy probiotics microorganisms, thus creating a beneficial gastrointestinal system, resulting in positive effects to the host’s health [188,192]. Symbiotics should be created by selecting an appropriate combination of probiotics and prebiotics, in order to promote the growth and survival of probiotics in the intestinal tract. Furthermore, the symbiotic formula should be more effective compared to the activity of the individual components [205]. Some studies report the beneficial effects of symbiotic supplementation in biochemical and histological features of NAFLD [206,207,208,209,210,211,212]. Malaguarnera et al. found that the combination of *B. longum* and FOS, together with lifestyle modification, reduces AST, TNFα, and C-reactive protein (CRP) levels, HOMA index and serum endotoxin, as well as decreases inflammation and steatosis, in 66 NASH patients [206]; moreover, a randomized, double-blind, placebo-controlled clinical trial showed that supplementation of seven probiotic strains (*L. casei*, *L. rhamnosus*, *S. thermophilus*, *B. breve*, *L. acidophilus*, *B. longum*, and *L. bulgaricus*) and FOS significantly reduced liver enzymes (ALT, AST, and GGT), and inflammatory markers (TNFα, CRP, and total nuclear factor k-B p65) in 52 patients with NAFLD [207]. Another randomized, double-blind, placebo-controlled clinical trial reported that the combination of dietary fiber and *L. reuteri* reduced fibrosis, hepatic steatosis, and serum levels of inflammatory markers in 50 lean patients with NAFLD [208]. Finally, in a recent clinical trial (the INSYTE study), Scorletti (2020). observed that *Bifidobacterium animalis subsp. lactis BB-12* and FOS alter fecal microbiome, but do not reduce liver fat content and markers of liver fibrosis [209]. Symbiotics have the ability to modulate the GM of the elderly [213,214,215]. Two double-blind, placebo-controlled clinical trials report that the mixture of *Bifidobacterium bifidum BB-02*, *Bifidobacterium lactis BL-01*, and inulin, as well as mixture of *Lactobacillus acidophilus NCFM* and lactitol can increase the growth of *Bifidobacteria* and *Lactobacilli* [213,215]; in addition, another clinical trial shows that the combination of *Bifidobacterium longum* and inulin increased the number of *Actinobacteria* and *Firmicutes*, and decreased *Proteobacteria* [214]. Interestingly, Marìa Juàrez-Fernàndez et al. observed the beneficial effect of the symbiotic combination of the NGP *A. muciniphila* and quercetin on NAFLD, by modulating GM composition and bile acid metabolism [216].

*Fecal microbiota transplant (FMT)* is the process by which fecal material from healthy donors is inserted into the intestine of patients with an altered GM, in order to restore it to a stable state and thus treat specific diseases related to dysbiosis [217]. At present, FMT has been used successfully in patients with recurrent *Clostridum difficile* infection, metabolic syndrome, inflammatory bowel syndrome, and obesity [218], and could become an effective therapeutic method for the treatment of NAFLD. It has been demonstrated that restoration of a healthy GM with FMT treatment alleviated steatohepatitis in HFD model mice [219], and restored portal hypertension, insulin resistance, and endothelial dysfunction in NASH model rats [220]. To date, limited human studies have been conducted, and not all have shown beneficial effects of FMT in the treatment of NAFLD. For instance, a double blind, randomized, controlled proof-of-principle study reported that allogenic donor FMT in individuals with hepatic steatosis produced beneficial changes in hepatic gene expression and in metabolites involved in inflammation and lipid metabolism [221]; in addition, another randomized, controlled trial shows that allogenic FMT in patients with NAFLD can reduce small intestinal permeability, but do not improve insulin resistance nor reduce hepatic fat fraction [222].

## 9. Conclusions

NAFLD is a common liver disease, especially widespread among elderly people with metabolic disorders, which is characterized by excessive fat accumulation in hepatocytes. Several experimental studies conducted both in aged animals, (in which the pathological symptoms of the disease are induced by high-fat and MCD diets) and in adult patients with NAFLD, have highlighted the presence of an altered GM, compared to the one observed in healthy people. In elderly people, the GM is characterized by a particular microbial signature (increase in Gram-negative bacteria and pathobionts, with a consequent release of endotoxins and LPS, and reduction in Gram-positive microorganisms), and this altered GM seems to play a relevant role in promoting the pathogenesis of NAFLD. In fact, the intestinal dysbiosis, together with a high level of OS, determines an increase in the intestinal permeability with a consequent release of ROS, endotoxins, and LPS into the bloodstream. All together, these events lead to an increased susceptibility to develop the disease and favor its progression into NASH. Therefore, as several experimental studies and clinical trials highlight, the restoration of the altered GM to a healthy state could be a new beneficial weapon to manage NAFLD. Probiotics supplementation, alone or in combination with NAFLD traditional treatments, could then represent a new therapeutic approach capable of reinstating a balanced intestinal flora, even if their synergic action is not yet well known. Indeed, although probiotics have been used for decades to prevent or treat some disorders, to date, their efficacy in counteracting or alleviating NAFLD has not yet been fully explored. In fact, although promising, both preclinical researches and randomized controlled trials are still few to demonstrate therapeutic efficacy in NAFLD management. Moreover, more studies are required, on one side, to better clarify the precise role of the altered GM in the pathogenesis of this hepatic disease, and on the other, to find the most effective probiotic strains that can be used, the dosage to be administered, and the duration of the treatment.

## Figures and Tables

**Table 1:** Use of probiotics in several disorders.

Disease	Probiotic	Reference
Acute diarrhea	*Lactobacillus rhamnosus GG*	[110]
Allergic rhinitis	*Lactobacillus acidophilus L-92*	[111]
Antibiotic-associated diarrhea	*Saccharomyces boulardii*	[112]
Asthma	*Enterococcus faecalis FK-23*	[113]
Atopic dermatitis	*Lactobacillus paracasei*, and *Lactobacillus fermentum*	[114]
Atopic eczema	Mixture (*Bifidobacterium bifidum*, *Bifidobacterium lactis*, and *Lactobacillus acidophilus*)	[115]
Bacterial vaginosis	*Saccharomyces cerevisiae Lactobacillus acidophilus*, *Lactobacillus rhamnosus GR-1*, and *Lactobacillus fermentum RC-14*	[116] [117]
Cardiovascular disorder	*Lactobacillus rhamnosus GG*	[118]
Chronic diarrhea	*Lactobacillus plantarum CCFM1143*	[119]
Colon cancer	*Lactobacillus rhamnosus Lactobacillus acidophilus*, Mixture (*Bifidobacteria bifidum*, and *Bifidobacteria infantum*)	[120] [121]
Crohn’s disease	*Escherichia coli Nissle 1917 Saccharomyces boulardii*	[122] [123]
Diabetes	*Lactobacillus acidophilus*	[124]
Diarrhea	*Bifidobacterium bifidum FSDJN705*, and *Bifidobacterium breve FHNFQ23M3*	[125]
Gastroenteritis	*Lactobacillus F19*	[126]
Hypercholesterolemia	*Enterococcus faecium M-74*	[127]
Lactose intolerance	*Lactobacillus acidophilus DDS-1*	[128]
Liver disorder	*Escherichia coli Nissle* VSL*#3*	[129] [130]
Metabolic disorder	*Bifidobacterium adolescentis Z25*	[131]
Obesity	*Lactobacillus plantarum K50*	[132]
Urinary tract infections	*Lactobacillus rhamnosus GR-a* and *Lactobacillus reuteri RC-14*	[133]

**Table 2:** List of probiotics strains commonly used.

*Lactobacilli*	*Bifidobacteria*	*Saccharomyces*	*Other species*
*L. acidophilus*	*B. adolescentis*	*S. boulardii*	*Bacillus subtilis*
*L. casei*	*B. animalis*	*S. cerevisiae*	*Enterococcus faecalis*
*L. crispatus*	*B. bifidum*		*Escherichia coli*
*L. fermentum*	*B. breve*		*Lactococcus lactis*
*L. gallinarum*	*B. infantis*		*Streptococcus thermophilus*
*L. gasseri*	*B. longum*		
*L. helveticus*			
*L. johnsonii*			
*L. lactis*			
*L. paracasei*			
*L. plantarum*			
*L. reuteri*			
*L. rhamnosus*			

**Table 3:** Effects of probiotics treatment in animals and human experimental studies.

Probiotic	Model	Diet	Duration	Treatment Effects	Reference
***Lactobacillus rhamnosus GG***	Mice	High-fructose diet-induced NAFLD	8 weeks	1. Improvement of the accumulation of fat in the liver 2. Reduction of liver inflammation (↓TNFα, ↓IL-8R, ↓IL-1β), as well as steatosis 3. Increase in gut beneficial bacteria 4. Restoration of tight junction proteins, resulting in gut barrier function amelioration	[167]
***Lactobacillus rhamnosus GG*** **and *Lactobacillus plantarum WCFS1***	Sprague-Dawley rats	High-fat diet-induced NAFLD	21 weeks	1. Reduction of gut endotoxemia level, as well as the expression of inflammatory cytokines 2. Amelioration of GM and intestinal barrier function 3. Increase in CYP7A1 and LDL-R, resulting in improvement of lipid metabolism and insulin resistance	[183]
***Bifidobacterium infantis*** **, *Lactobacillus acidopilus*, *Bacillus cereus***	Rats	High-fat/high-sucrose diet-induced NAFLD	12 weeks	1. Downregulation of LPS/TLR4 signaling pathway, resulting in slowing the progression of NAFLD 2. Improvement of GM dysbiosis and the intestinal barrier function 3. Reduction of body weight 4. Decrease in TNFα, and IL-18 expression, as well as ALT, AST, GGT, and ALP activities	[171]
***Lactobacillus plantarum ATG-K2*** **and *ATG-K6***	Wistar rats	High-fat and fructose-diet-induced NAFLD	8 weeks	1. Modulation of GM 2. Downregulating of de novo lipogenesis-associated genes 3. Reduction of body weight and hepatic lipid accumulation 4. Increasing of antioxidant enzymes (SOD, GPx, CAT), and decreasing of ALT and AST serum levels	[184]
***Bifidobacterium animalis*** **subsp. *Lactis V9***	Wistar rats	High-fat diet-induced NAFLD	9 weeks	1. Decrease in ALT, AST, TLR4, and TLR9 levels, resulting in alleviation of hepatic steatosis and liver damage 2. Reduction of serum glucose level, as well as hepatic triglycerides and free fatty acids accumulation 3. Restoration of hepatic phosphorylated-AMPK and PPAR-α levels, and reduction of SREBP-1c and FAS expression 4. Attenuation of liver inflammation, by inhibiting inflammatory cytokines synthesis (IL-6, IL-1β, TNFα)	[185]
***Lactobacillus acidophilus La5*** **, *Bifidobacterium lactis Bb12***	72 NAFLD patients		8 weeks	1. Decreasing of ALT and AST activity 2. Reduction of triglycerides and low-density lipoprotein cholesterol serum levels, as well as total cholesterol	[186]
**Multiprobiotic “Lactocare” (*L. casei*, *L. acidophilus*, *L. rhamnosus*, *L. bulgaricus*, *B. breve*, *B. longum*, *Streptococcus thermophilus*)**	42 NAFLD patients		8 weeks	1. Decrease in TNFα and IL-6 expression, as well as FBS and insulin	[178]
**Probiotics mixture (*Bifidobacterium*, *Lactobacillus*, and *Enterococcus*; *Bacillus subtilis* and *Enterococcus*)**	200 NAFLD patients		1 month	1. Improvement of GM composition, by inhibiting TNFα expression and ameliorating adiponectin level 2. Decrease in ALT and AST serum levels 3. Amelioration of lipid metabolism and fatty liver	[157]
**Multiprobiotic “Symbiter” (*Bifidobacterium*, *Lactobacillus*, *Lactococcus*, *Propionibacterium*, *Acetobacter*)**	58 NAFLD patients		8 weeks	1. Reduction of liver fat (↓total cholesterol and ↓triglycerides) 2. Decreasing of AST and GGT activity, as well as TNFα and IL-6 expression	[182]
***Lactobacillus paracasei DSM 24733*** **, *Lactobacillus plantarum DSM 24730*, *Lactobacillus acidophilus DSM 24735* and *Lactobacillus delbrueckii* subsp. *bulgaricus DSM 24734*, *Bifidobacterium longum DSM 24736*, *Bifidobacterium infantis DSM 24737*, *Bifidobacterium breve DSM 24732*, and *Streptococcus thermophilus DSM 24731***	30 NAFLD patients		12 months	1. Improvement of liver histology 2. Reduction in steatohepatitis 3. Decrease in ALP, AST, and ALT activity, as well as endotoxins, TNFα, IL-1β, and IL-6 levels	[187]

Abbreviations: ALP: alkaline phosphatase; ALT: alanine aminotransferase; AMPK: AMP-activated protein kinase; AST: aspartate aminotransferase; CAT: catalase; CYP7A1: cholesterol 7 α-hydroxylase; FAS: lipogenic enzyme fatty acid synthase; FBS: fasting blood sugar; GPx: glutathione peroxidase; GGT: gamma glutamyl transferase; IL-1β: interleukin 1β; IL-6: interleukin 6; IL-8R: interleukin 8 receptor; IL-18: interleukin 18; LDL-R: low-density lipoprotein receptor; LPS/TLR4: lipopolysaccharide/toll-like receptor 4; PPAR-α: peroxisome proliferator-activated receptor α; SOD: superoxide dismutase; SREBP-1c: sterol-regulatory element binding protein-1c; TLR9: toll-like receptor 9; TNFα: tumor necrosis factor α.
